# Improved PPP Ambiguity Resolution Considering the Stochastic Characteristics of Atmospheric Corrections from Regional Networks

**DOI:** 10.3390/s151229772

**Published:** 2015-11-30

**Authors:** Yihe Li, Bofeng Li, Yang Gao

**Affiliations:** 1 Department of Geomatics Engineering, University of Calgary, Calgary, AB T2N 1N4, Canada; ygao@ucalgary.ca; 2 College of Surveying and Geo-Informatics, Tongji University, Shanghai 200092, China; bofeng_li@tongji.edu.cn

**Keywords:** global navigation satellite system (GNSS), regional reference network, regional augmented PPP, ambiguity resolution (AR), atmospheric stochastic model

## Abstract

With the increased availability of regional reference networks, Precise Point Positioning (PPP) can achieve fast ambiguity resolution (AR) and precise positioning by assimilating the satellite fractional cycle biases (FCBs) and atmospheric corrections derived from these networks. In such processing, the atmospheric corrections are usually treated as deterministic quantities. This is however unrealistic since the estimated atmospheric corrections obtained from the network data are random and furthermore the interpolated corrections diverge from the realistic corrections. This paper is dedicated to the stochastic modelling of atmospheric corrections and analyzing their effects on the PPP AR efficiency. The random errors of the interpolated corrections are processed as two components: one is from the random errors of estimated corrections at reference stations, while the other arises from the atmospheric delay discrepancies between reference stations and users. The interpolated atmospheric corrections are then applied by users as pseudo-observations with the estimated stochastic model. Two data sets are processed to assess the performance of interpolated corrections with the estimated stochastic models. The results show that when the stochastic characteristics of interpolated corrections are properly taken into account, the successful fix rate reaches 93.3% within 5 min for a medium inter-station distance network and 80.6% within 10 min for a long inter-station distance network.

## 1. Introduction

Precise Point Positioning (PPP) has the capability of providing centimeter or even millimeter positioning accuracy using a single dual-frequency global navigation satellite system (GNSS) receiver [1]. PPP has been demonstrated to be a powerful tool to support a wide range of applications, including precise positioning [2], atmospheric water vapor sensing [3,4], earthquake and tsunami monitoring [5], ocean-tide measurement [6], precision agriculture [7] as well as many other remote sensing applications [8].

Traditional PPP is based on real-valued ambiguity solutions and typically suffers from long convergence times. In order to shorten the convergence time, ambiguity resolution (AR) is necessary, and several approaches have been developed for this purpose in recent years [9,10,11,12]. A key element for PPP AR is the estimation of the fractional cycle biases (FCBs) so as to recover the integer feature of ambiguities [13]. If a global network is used, the ionosphere-free (*L_IF_*) ambiguities can be decomposed into wide-lane (WL) and narrow-lane (NL) ambiguities. However, the process still needs a few tens of minutes in order to reliably fix the ambiguities [14]. If a regional network is available, the atmospheric (ionosphere and troposphere) corrections can be precisely derived from the network [15]. In such cases, an innovative technology called regional augmented PPP can be used so that the integer ambiguity resolution can be significantly accelerated using the precisely predicted ionospheric delays [16].

The essential difference between PPP and network RTK (NRTK) stems from the distinct types of corrections generated at the network level: processing of network data into corrections in the state space representation (SSR) enables PPP implementation, while the network correction given in the observation state representation (OSR) leads to NRTK implementation. Compared to NRTK, several major advantages of regional augmented PPP can be identified [17]. First, the bandwidth can be significantly reduced, because the dynamics of different state corrections can be utilized to optimize the bandwidth. Second, SSR better represents the associated errors. The interpolation of the different physical parameters can use different and optimized mathematical models as well as the stochastic properties of the parameters. Third, SSR can be made independent from the regional reference network. The role of the regional network for augmented PPP is to generate precise atmospheric corrections to reduce the convergence time. If such regional networks are not available, the regional augmented PPP is reduced to the global PPP scenario, which can still do precise positioning, although it requires a longer convergence period.

The proper determination of a mathematical model for atmospheric corrections is essential for regional augmented PPP, which includes both functional and stochastic models. As to the functional model, several examples have been developed, including the linear combination method (LCM) [18], linear interpolation method (LIM) [19], distance-based linear interpolation method (DIM) [20], lower-order surface model (LSM) [21], least-squares collocation method (LSCM) [22], and modified linear combination method (MLCM) [16]. However, very limited work has been done so far on stochastic models of atmospheric corrections. In fact, an incorrect stochastic model will not only result in incorrect solutions, but also slow down the convergence, even if a proper functional model is applied. This fact highlights a need to investigate the impact of the stochastic model on the regional augmented PPP AR and to develop some way of determining the stochastic model of atmospheric corrections generated by the regional network.

Several studies have considered the stochastic characteristics of atmospheric corrections. For networks with inter-station distances around 50 km or less, the atmospheric corrections generated from the network are usually treated as deterministic quantities without any consideration of their uncertainties [23], which, however, would result in model errors. Since the estimated atmospheric corrections from the reference stations are stochastic rather than deterministic in nature, the obtained interpolated corrections based on these estimates are stochastic variables as well, which should be taken into account or the solutions would be too optimistic [24]. For networks with inter-station distances greater than 50 km, the atmospheric corrections generated from the network are usually treated as stochastic with empirical uncertainties [25,26,27]. However, these empirical values often fail to capture the variation of the atmospheric behavior in high spatiotemporal resolutions. When the values are too small, the ambiguity float solution will be biased and the fixed ambiguity will not be reliable; when the values are too large, the efficiency of the ambiguity fixing becomes lower, *i.e.*, the ambiguities that can be confidently fixed are left unfixed [23].

This paper aims to improve the performance of regional augmented PPP ambiguity resolution by strengthening its float solutions with properly estimated stochastic characteristics of the atmospheric corrections generated from the regional network. The method starts with the generation of the satellite FCBs, ionospheric and tropospheric delays for the user stations based on the observations of the regional reference network. Instead of determining the empirical variances of atmospheric corrections as a linear function of the average distance amongst the reference stations as in the traditional strategy, we determine the variances of interpolated atmospheric corrections by considering both their random uncertainties and their discrepancy against the actual atmospheric correction at the user station. The variance of the first component (random) is estimated by the error propagation law, while the second component (discrepancy) comes from the regional network. The interpolated atmospheric corrections are used as the pseudo-observations together with the estimated variance-covariance matrix to describe the strength level of the constraint. Two experiments from different sizes of networks are processed to demonstrate the superior PPP AR performance of the proposed method.

## 2. Network Data Processing

### 2.1. Network undifferenced Observation Model

Since the coordinates of all network reference stations and the precise satellite orbits are given, the single-epoch undifferenced GPS code and phase observations on the *j*th frequency can be written as:

(1) $$\begin{array}{l} P_{r,j}=e_{n}dt^{r}-dt^{s}+g\tau +\mu_{j}\iota +e_{n}b_{P_{j}}^{r}-b_{P_{j}}^{s}+\varepsilon_{P_{j}}\\ L_{r,j}=e_{n}dt^{r}-dt^{s}+g\tau -\mu_{j}\iota -\lambda_{j}N_{r,j}+e_{n}b_{L_{j}}^{r}-b_{L_{j}}^{s}+\varepsilon_{L_{j}} \end{array}$$

where *r* denotes the receiver; j  denotes the frequency. Assuming that *n* satellites are simultaneously tracked, $P_{r,j}\;$ and $L_{r,j}$ are the undifferenced code and phase observation vector corrected with the geometric distance computed by using the known receiver and satellite coordinates; $dt^{r}\;$ and $dt^{s}\;$ are the receiver clock and satellite clock vector; τ  is the Zenith Tropospheric Delay (ZTD) with its mapping matrix *g*; ι  is the first-order ionospheric delay on frequency *L_1_* with $\mu_{j}=f_{1}^{2}/f_{j}^{2}$; $\lambda_{j}$ is the carrier phase wavelength, $N_{r,j}$ is the integer ambiguity vector (cycle); $e_{n}$ = ${\left[1\;\ldots 1\right]}^{T}$; $b_{P_{j}}^{r}$ and $b_{P_{j}}^{s}$ are the receiver code bias and satellite code bias vector; $b_{L_{j}}^{r}$ and $b_{L_{j}}^{s}$ are the receiver phase bias and satellite phase bias vector; $\varepsilon_{P_{j}}$ and $\varepsilon_{L_{j}}$ are the code and phase observation errors including multipath and noises.

We define:

(2) $$B_{r,j}=N_{r,j}-e_{n}b_{L_{j}}^{r}+b_{L_{j}}^{s}$$

where $B_{r,j}$ is the comprehensive ambiguity without integer property.

Collecting single-epoch dual-frequency observations $y_{r}={\left[\begin{array}{cc} \begin{array}{cc} P_{r,1}^{T} & P_{r,2}^{T} \end{array} & \begin{array}{cc} L_{r,1}^{T} & L_{r,2}^{T} \end{array} \end{array}\right]}^{T}$, the stochastic model of $y_{r}$ can be specified by $Q_{y_{r}}=Q_{f}⊗Q_{s}$ where ⊗ is Kronecker product operator; $Q_{f}=\text{blockdiag}\left(Q_{P},\;Q_{L}\right)$ captures the frequency-specific precision contribution with $Q_{L}=diag\left(\begin{array}{cc} \sigma_{L_{r,1}}^{2} & \sigma_{L_{r,2}}^{2} \end{array}\right)$ and $Q_{P}=diag\left(\begin{array}{cc} \sigma_{P_{r,1}}^{2} & \sigma_{P_{r,2}}^{2} \end{array}\right)$, $\sigma_{L_{r,j}}^{2}$ and $\sigma_{P_{r,j}}^{2}$ are variances of the undifferenced phase and code on frequency *j*. $Q_{s}$ is the satellite elevation-dependent cofactor matrix of the undifferenced observations, which can be expressed as [28]:

(3) $$Q_{s}=diag\left(\begin{array}{ccc} q_{s}^{1} & \cdots & q_{s}^{n} \end{array}\right)\;\{\begin{array}{cc} q_{s}^{i}=1 & \theta \geq 30^{o}\\ q_{s}^{i}=0.5/\sin\theta & \theta <30^{o} \end{array},i=1\cdots n$$

where θ is satellite elevation angle; $q_{s}^{i}\;$ is cofactor of the undifferenced observations.

In order to remove ionospheric delays, ionosphere-free observations are usually used for PPP. The ionosphere-free observation can be expressed as follows:

(4) $$\begin{array}{l} L_{r,IF}+dt_{P_{IF}}^{s}=e_{n}dt_{P_{IF}}^{r}+g\tau -\lambda_{IF}B_{r,IF}+\varepsilon_{L_{IF}}\\ P_{r,IF}+dt_{P_{IF}}^{s}=e_{n}dt_{P_{IF}}^{r}+g\tau +\varepsilon_{P_{IF}}\\ \lambda_{IF}B_{r,IF}=\alpha_{IF}\lambda_{1}B_{r,1}+\beta_{IF}\lambda_{2}B_{r,2}=\lambda_{NL}B_{r,NL}+\beta_{IF}\lambda_{2}B_{r,WL} \end{array}$$

where $\;P_{r,IF}\text{ and }L_{r,IF}$ are the code and phase ionosphere-free observation; $\alpha_{IF}=f_{1}^{2}/\left(f_{1}^{2}-f_{2}^{2}\right);$ $\beta_{IF}=f_{2}^{2}/\left(f_{1}^{2}-f_{2}^{2}\right);\;\lambda_{IF}$ is the ionosphere-free wavelength.

Ambiguity resolution is conducted first in wide-lane, and then in narrow-lane. The float WL ambiguities $B_{WL}$ can be calculated simply by taking the time average of the Melbourne and Wübbena (MHW) combination [29,30,31] of the dual-frequency phase and code observations. The ionosphere-free ambiguities are estimated from the PPP solution. The float NL ambiguities $B_{NL}$ are derived from Equation (4) with the fixed WL ambiguity and the float ionosphere-free ambiguity $B_{IF}$ . To recover the integer nature of ambiguities, the satellite WL and NL FCBs ( $b_{MHW}^{s},b_{NL}^{s}\;)\;$ must be estimated from the reference network.

Meanwhile the ZTD  τ  and its variance can be also obtained from the PPP solution. The variance of ZTD $\sigma_{\tau }^{2}$ at the reference station can be expressed as:

(5) $$\sigma_{\tau }^{2}=\left[\begin{array}{cccc} \alpha_{IF} & -\beta_{IF} & \alpha_{IF} & -\beta_{IF} \end{array}\right]Q_{f}{\left[\begin{array}{cccc} \alpha_{IF} & -\beta_{IF} & \alpha_{IF} & -\beta_{IF} \end{array}\right]}^{T}q_{\tau }$$

where $q_{\tau }\;$ is the cofactor of ZTD extracted from the cofactor matrix.

### 2.2. FCB Estimation and Ambiguity Resolution from Network

Let us assume that we have a network of *n* stations tracking *m* satellites. The float undifferenced ambiguities at each station are estimated as *B_i_*. For all the float ambiguity, we have an observation as follows:

(6) $$\left[\begin{array}{c} B_{1}\\ B_{2}\\ \vdots \\ \vdots \\ B_{n} \end{array}\right]=\left[\begin{array}{ccccccc} I & & & & & R_{1} & S_{1}\\ & I & & 0 & & R_{2} & S_{2}\\ & & I & & & \vdots & \vdots \\ & 0 & & I & & \vdots & \vdots \\ & & & & I & R_{n} & S_{n} \end{array}\right]\left[\begin{array}{c} n_{1}\\ n_{2}\\ \vdots \\ n_{n}\\ b^{r}\\ b^{s} \end{array}\right]$$

In matrix *R_i_* all elements of one column are –1 and all other entries are zero. For matrix *S_i_* each line has one element of 1, the other entries are zero. *n_i_* is the undifferenced integer ambiguity vector for station *i*.

Under the condition that all the integer ambiguities are known and that one receiver FCB is fixed to zero, the satellite and receiver FCBs can be estimated by means of a least square from the following observation Equation (7):

(7) $$\left[\begin{array}{c} B_{1}-n_{1}\\ B_{2}-n_{2}\\ \vdots \\ \vdots \\ B_{n}-n_{n} \end{array}\right]=\left[\begin{array}{cc} R_{1} & S_{1}\\ R_{2} & S_{2}\\ \vdots & \vdots \\ \vdots & \vdots \\ R_{n} & S_{n} \end{array}\right]\left[\begin{array}{c} b^{r}\\ b^{s} \end{array}\right]$$

We assume that the receiver FCB at the arbitrarily selected station is zero. Then the nearest integers of the ambiguities at this station are the integer ambiguities and the fractional parts are estimates of the corresponding satellite FCBs. When applying these satellite FCBs to the common satellites of the next station, the corrected ambiguities should have a very similar fractional part. The mean fractional parts of all the common satellites correspond to the receiver FCB. With this receiver FCB, FCBs of the newly appearing satellites at the station can be estimated. Repeating this procedure for all stations, we can obtain the approximate FCBs for all receivers and satellites.

After correcting the ambiguities with the FCBs, the integer property of ambiguity can be recovered, thus ambiguity-fixing can be attempted. By replacing integer ambiguity parameters with their fixed values in Equation (7), the remaining parameters can be estimated. The FCB estimates are improved and will in turn help to resolve more integer ambiguities. The above procedure can be done iteratively until no more integer ambiguities can be fixed.

The approach described above is first applied to all the float undifferenced WL ambiguities, so that the WL FCBs and integer WL ambiguities are estimated. With the integer WL ambiguities estimated, the float NL ambiguities are derived from the ionosphere-free ambiguities. Afterwards, the same approach is used to estimate the NL FCBs from the float NL ambiguities. For more details about the above FCB and ambiguity estimation process, one refers to [32].

## 3. Atmospheric Correction Interpolation

Once the WL and NL FCBs are determined, L1 and L2 FCBs can be also recovered as follows:

(8) $$\begin{array}{l} {\tilde{b}}_{L1}^{s}=\frac{\beta_{WL}b_{NL}^{s}-\beta_{IF}b_{MHW}^{s}}{\alpha_{IF}\beta_{WL}-\alpha_{WL}\beta_{IF}},{\tilde{b}}_{L1}^{r}=\frac{\beta_{WL}b_{NL}^{r}-\beta_{IF}b_{MHW}^{r}}{\alpha_{IF}\beta_{WL}-\alpha_{WL}\beta_{IF}}\\ {\tilde{b}}_{L2}^{s}=\frac{\alpha_{WL}b_{NL}^{s}-\alpha_{IF}b_{MHW}^{s}}{\alpha_{IF}\beta_{WL}-\alpha_{WL}\beta_{IF}},{\tilde{b}}_{L2}^{r}=\frac{\beta_{WL}b_{NL}^{r}-\beta_{IF}b_{MHW}^{r}}{\alpha_{IF}\beta_{WL}-\alpha_{WL}\beta_{IF}} \end{array}$$

Thus, we can derive the ionospheric delays based on the phase observations as follows:

(9) $$\tilde{\iota }=\beta_{IF}\left(L_{r,1}-L_{r,2}+\lambda_{1}N_{r,1}+{\tilde{b}}_{L_{1}}^{s}-{\tilde{b}}_{L_{1}}^{r}-\lambda_{2}N_{r,2}-{\tilde{b}}_{L_{2}}^{s}+{\tilde{b}}_{L_{2}}^{r}\right)$$

As stated in Teunissen and Khodabandeh [33], the ionospheric delays derived from the code and phase observations actually have identical interpretation as $\tilde{\iota }=\;\iota +\beta_{IF}\left(b_{P_{1}}^{s}-b_{P_{2}}^{s})-\beta_{IF}e_{n}(b_{P_{1}}^{r}-b_{P_{2}}^{r}\right)$. Thus, the ionospheric delay derived from ambiguity-fixed phase observation can also be used to correct the code observations at the user stations.

The slant ionospheric and ZTD corrections at reference stations are used to interpolate the atmospheric correction at user station by using inverse distance-based interpolation [26]:

(10a) $$\tau_{u}=\sum_{i=1}^{i=n_{r}}\frac{1}{D_{i}}\tau_{i}/\sum_{i=1}^{i=n_{r}}\frac{1}{D_{i}}$$

where $\tau_{i}$ and $\tau_{u}$ are the ZTD correction at reference station i and user station u, $D_{i}$ is the distance from reference station i to user station. The biased ionospheric correction derived from code and phase observations for user station u can be interpolated as:

(10b) $${\tilde{\iota }}_{u}=\sum_{i=1}^{i=n_{r}}\frac{1}{D_{i}}\left(\iota_{i}-\beta_{IF}e_{n}\left(b_{P_{2}}^{i}-b_{P_{1}}^{i}\right)\right)/\sum_{i=1}^{i=n_{r}}\frac{1}{D_{i}}+\beta_{IF}\left(b_{P_{1}}^{s}-b_{P_{2}}^{s}\right)$$

After interpolation, the atmospheric correction for the user stations can be defined as $z_{u}={\left[\begin{array}{cc} \tau_{u} & {\tilde{\iota }}_{u}\; \end{array}\right]}^{T}$.

## 4. Stochastic Model of Interpolated Atmospheric Corrections

The covariance matrix of interpolated atmospheric corrections has been usually determined using empirical functions. Taking the ionospheric delay as the example, it is common that the STD of the ionospheric corrections is modeled as a linear function of the baseline length *l* [34]:

(11) $$\sigma_{\iota }=\beta l$$

Similarly, the linear function can also be employed to determine the atmospheric stochastic model for network augmented PPP because undifferenced and double-differenced data processing are only different implementations (utilization) of the network data. Odijk [35] suggested β = 0.57 mm/km in quiet ionospheric conditions. Liu and Lachapelle [36] suggested  β = 0.74 and β = 1.04 mm/km in time of low and high ionospheric activities. However, these empirical values are still difficult to capture the spatial and temporal variations of the ionospheric activities. To overcome this limitation, the stochastic model of the atmospheric corrections should be established by fully exploiting the network information.

The uncertainties of the interpolated ionospheric and ZTD corrections theoretically depend on two uncorrelated error sources. One source is the error caused by the multipath and noises in the estimated corrections at the reference stations, called Source I error in the sequel. The covariance matrix corresponding to this error source is expressed as $cov\;{\left(z_{u}\right)}_{p}$. The other source is the modeling error of the atmospheric corrections over the reference network, called Source II error in the sequel. The corresponding covariance matrix is expressed as $cov\;{\left(z_{u}\right)}_{m}$. In the following, a method to the determination of the stochastic model for the interpolated atmospheric corrections will be described.

Based on random error propagation law, the covariance matrix of the interpolated atmospheric corrections *cov* ($z_{u}$) can be expressed as:

(12) $$\mathrm{cov}\left(z_{u}\right)=\mathrm{cov}{\left(z_{u}\right)}_{p}+\mathrm{cov}{\left(z_{u}\right)}_{m}$$

Due to the very weak correlation between the interpolated ZTD and the ionospheric errors, see e.g., Figure 3 and further explanations given later in Section 6, the covariance matrices $cov\;{\left(z_{u}\right)}_{p}$ and $cov\;{\left(z_{u}\right)}_{m}$ can therefore be simplified to a diagonal matrix as follows:

(13a) $$\;cov\;{\left(z_{u}\right)}_{p}=diag\left(\sigma_{\tau_{p}}^{2},\sigma_{{\tilde{\iota }}_{p}^{1}}^{2},\ldots ,\sigma_{{\tilde{\iota }}_{p}^{n}}^{2}\right)=diag\left(\sigma_{\tau_{p}}^{2},Q_{{\tilde{\iota }}_{p}}\right)$$

(13b) $$\;cov\;{\left(z_{u}\right)}_{m}=diag\left(\sigma_{\tau_{m}}^{2},\sigma_{{\tilde{\iota }}_{m}^{1}}^{2},\ldots ,\sigma_{{\tilde{\iota }}_{m}^{n}}^{2}\right)=diag\left(\sigma_{\tau_{m}}^{2},Q_{{\tilde{\iota }}_{m}}\right)$$

where $\sigma_{\tau_{p}}^{2},\sigma_{{\tilde{\iota }}_{p}^{1}}^{2},\ldots ,\sigma_{{\tilde{\iota }}_{p}^{n}}^{2}$ are the variances of the Source I error of the interpolated ZTDs and the slant ionospheric delays$,\;Q_{{\tilde{\iota }}_{p}}=diag\left(\sigma_{{\tilde{\iota }}_{p}^{1}}^{2},\ldots ,\sigma_{{\tilde{\iota }}_{p}^{n}}^{2}\right)$; $\sigma_{\tau_{m}}^{2},\sigma_{{\tilde{\iota }}_{m}^{1}}^{2},\ldots ,\sigma_{{\tilde{\iota }}_{m}^{n}}^{2}$ are the variances of the Source II error of the interpolated ZTDs and slant ionospheric delays$,Q_{{\tilde{\iota }}_{m}}=diag\left(\sigma_{{\tilde{\iota }}_{m}^{1}}^{2},\ldots ,\sigma_{{\tilde{\iota }}_{m}^{n}}^{2}\right)$.

The covariance matrix for the Source I error can be derived based on Equations (5), (10a) and (10b):

(14) $$\mathrm{cov}{\left(z_{u}\right)}_{p}=\frac{\sum_{i=1}^{i=n_{r}}\left(\frac{1}{D_{i}^{2}}\mathrm{cov}\left(z_{i}\right)\right)}{{\left(\sum_{i=1}^{i=n_{r}}\frac{1}{D_{i}}\right)}^{2}}=\frac{\sum_{i=1}^{i=n_{r}}\left(\frac{1}{D_{i}^{2}}{\left(C\left(Q_{f}⊗Q_{s}\right)C^{T}\right)}_{i}\right)}{{\left(\sum_{i=1}^{i=n_{r}}\frac{1}{D_{i}}\right)}^{2}}$$

where C is the coefficient matrix necessary for the calculation of the covariance matrix of the Source I error, which is expressed as follows:

(15) $$C=\left[\begin{array}{cccc} \alpha_{IF}e_{n}^{T}\sqrt{q_{\tau }Q_{s}^{-1}} & -\beta_{IF}e_{n}^{T}\sqrt{q_{\tau }Q_{s}^{-1}} & \alpha_{IF}e_{n}^{T}\sqrt{q_{\tau }Q_{s}^{-1}} & -\beta_{IF}e_{n}^{T}\sqrt{q_{\tau }Q_{s}^{-1}}\\ 0 & 0 & \beta_{IF}I_{n} & -\beta_{IF}I_{n} \end{array}\right]$$

Since the computational procedure is the same for ZTDs and slant ionospheric delays, only the variance determination for the Source II ionospheric error is described which includes a two-step procedure. The first step is to select a group of reference stations near the user station to interpolate the ionospheric errors at those selected stations and the second step is to apply those interpolated ionospheric errors at those selected stations to interpolate the ionospheric error at the user station. Details are given below:

*Step 1*: Determination of interpolated ionospheric errors at selected reference stations

**Figure 1:**
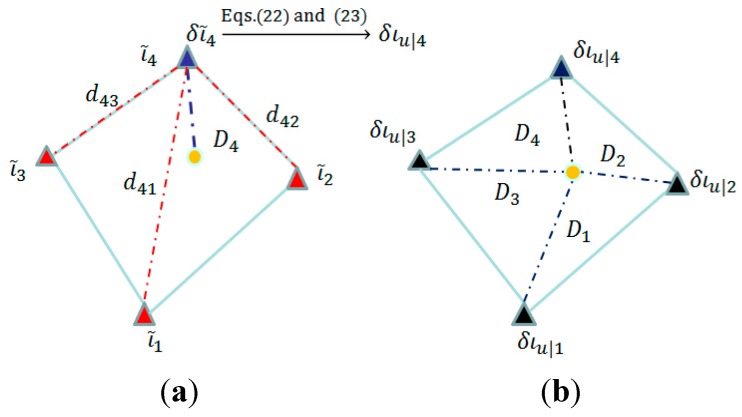
Atmospheric stochastic model estimated with the observations of a reference network. (**a**) determination of interpolated ionospheric errors at selected reference stations; (**b**) determination of variance for interpolated ionospheric error at user stations.

Shown in Figure 1a are reference stations selected nearby a user station. On the one hand, the ionospheric corrections at these reference stations have already been estimated using the network data. On the other hand, the ionopsheric correction at a selected station (e.g., the station in blue triangle in Figure 1a) can also be obtained through interpolation using those known ionospheric correction estimates at the remaining *n_r_* − 1 selected stations (in red triangles in Figure 1a). Their differences can then be used to compute the interpolated ionospheric correction error $\delta {\tilde{\iota }}_{s}$ at that selected station as follows:

(16) $$\delta {\tilde{\iota }}_{s}={\tilde{\iota }}_{s}-\frac{\sum_{i=1}^{i=n_{r}-1}\frac{1}{d_{si}}{\tilde{\iota }}_{i}}{\sum_{i=1}^{i=n_{r}-1}\frac{1}{d_{si}}}$$

where $d_{si}$ is the distance between a selected reference station whose ionospheric error is to be interpolated and other *n_r_* − 1 selected stations, ${\tilde{\iota }}_{s}$ and ${\tilde{\iota }}_{i}$ are the corresponding ionospheric correction estimate based on the network data for those selected stations.

The effect of the receiver biases can be removed by taking a weighted (a function of elevation angle) average of $\delta {\tilde{\iota }}_{s}$. The interpolated ionospheric error with receiver-bias removed is thus given as:

(17) $$\delta \iota_{s}=\delta {\tilde{\iota }}_{s}-e_{n}\sum_{j=1}^{j=n}\left(\delta {\tilde{\iota }}_{s}^{j}/q_{s}^{j}\right)/\sum_{j=1}^{j=n}\left(1/q_{s}^{j}\right)$$

As the ionospheric effects are proportional to the baseline length in real situation, the interpolated ionospheric error at a user station (yellow circle in Figure 1b) $\;\delta \iota_{u|s}$ can be obtained by prediction based on the interpolated ionospheric errors available at the selected stations as follows:

(18) $$\delta \iota_{u|s}=\frac{\bar{D}}{\bar{d}}\delta \iota_{s}$$

where $\bar{D}\;$ is the average distance between all selected stations and the user stations, $\bar{d}\;$ is the average distance between a selected reference station and other *n_r_* − 1 reference stations.

*Step 2*: Determination of variance for interpolated ionospheric error at user stations

Based on the *n_r_* interpolated ionospheric correction errors at all selected stations from Step 1, the variance of the ionospheric correction at a user station for satellite *j* at the *k*th epoch can be computed as follows:

(19) $$\sigma_{{\tilde{\iota }}_{m,k}^{j}}^{2}=\frac{\sum_{i=1}^{i=n_{r}}\frac{1}{D_{s}^{2}}\frac{\sum_{t=k-m+1}^{t=k}{\left(\delta \iota_{u|s}^{j}\right)}_{t}^{2}}{m}}{\sum_{i=1}^{i=n_{r}}\frac{1}{D_{i}^{2}}}$$

where *m* is the number of data epochs within the data window which should be reasonably selected. 

Finally, the covariance matrix $Q_{{\tilde{\iota }}_{m}}$ can be expressed as follows:

(20) $$Q_{{\tilde{\iota }}_{m}}=diag\left(\sigma_{{\tilde{\iota }}_{m}^{1}}^{2}\cdots \sigma_{{\tilde{\iota }}_{m}^{n}}^{2}\right)$$

where $\sigma_{{\tilde{\iota }}_{m}^{j}}^{2}$ is the variance of the slant ionospheric correction for satellite *j*.

The benefits of using the estimated variance for the user stations include: (1) the estimated variance adaptively varies with the inter-station distances of the network to describe the uncertainties of the ionospheric corrections, e.g., larger in the sparse network, and smaller when in a dense network; (2) the estimated variance can better capture the complicated ionospheric variability which differs from time to time during one day than empirical values; (3) In the kinematic mode, the estimated variance can reflect the ionospheric variability caused by spatial variations.

## 5. PPP AR with Atmospheric Corrections and Their Stochastic Model

Moving all the known terms to the left side, the satellite FCBs can be eliminated while the receiver code and phase biases remain which however can be assimilated by the receiver code and phase clocks. Therefore, such systematic biases have no effect on the ionospheric corrections at the user station. Finally the observation equations at the user station read as:

(21) $$\begin{array}{l} P_{u,j}-\mu_{j}{\tilde{\iota }}_{u}-g\tau_{u}+dt_{P_{IF}}^{s}=G\xi_{u}+e_{n}dt_{P_{u,j}}^{r}+g\delta \tau_{u}+\mu_{j}\delta \iota_{u}+\varepsilon_{P_{u,j}}\\ L_{u,j}+\mu_{j}{\tilde{\iota }}_{u}-g\tau_{u}+dt_{P_{IF}}^{s}+{\tilde{b}}_{L_{j}}^{s}=G\xi_{u}+e_{n}dt_{L_{u,j}}^{r}+g\delta \tau_{u}-\mu_{j}\delta \iota_{u}-\lambda_{j}N_{u,j}+\varepsilon_{L_{u,j}} \end{array}$$

where *G* is the design matrix of the unknown position increment vector $\xi_{u}$; $\delta \iota_{u}$ is the residual ionospheric delay; $\delta \tau_{u}$ is the residual ZTD.

The residual ionospheric delay $\delta \iota_{u}$ and ZTD $\delta \tau_{u}$ can be estimated with prior information. We form the following absolute constraint equation to speed up the ambiguity resolution:

(22) $$\varsigma_{k}^{0}=\varsigma_{k}+\varepsilon_{\iota_{k}}^{0},\;Q_{\varsigma_{k}^{0}}=diag\left(\sigma_{\tau_{u,k}}^{2},Q_{{\tilde{\iota }}_{u,k}}\right)$$

where $\varsigma_{k}={\left[\delta \begin{array}{cc} \tau_{k} & \delta \iota_{k}^{T} \end{array}\right]}^{T}$. The prior biases, $\varsigma_{k}^{0}={\left[\delta \begin{array}{cc} \tau_{k,0} & \delta \iota_{k,0}^{T} \end{array}\right]}^{T}$ with covariance matrix $Q_{\varsigma_{k}^{0}}$ can be applied by a set of pseudo observation equation. $\sigma_{\tau_{u,k}}^{2}$ and $Q_{{\tilde{\iota }}_{u,k}}$ are the predicted variance of ZTDs and the covariance matrix of the ionospheric delays. Both of them can be obtained from *cov* ($z_{u,k}$).

The sequential solutions of Kalman filter type will be derived based on the least squares criterion:

(23a) $$y_{k}=A_{k}x_{k}+\varepsilon_{k},\;Q_{y_{k}}$$

(23b) $$x_{k}=\Phi_{k,k-1}x_{k-1}+w_{k},\;Q_{w_{k}}$$

where $y_{k}={\left[\begin{array}{cc} \begin{array}{cc} P_{u,1,k}^{T} & P_{u,2,k}^{T} \end{array} & \begin{array}{cc} L_{u,1,k}^{T} & L_{u,2,k}^{T} \end{array} \end{array}\right]}^{T}$, $x_{k}={\left[\begin{array}{cc} \begin{array}{cc} \xi_{u,k}^{T} & dt_{u,k}^{r}\; \end{array} & \begin{array}{cc} \varsigma_{k}^{T} & \begin{array}{cc} N_{u,1}^{T} & N_{u,2}^{T} \end{array} \end{array} \end{array}\right]}^{T}$, $dt_{u,k}^{r}={\left[\begin{array}{cc} \begin{array}{cc} dt_{P_{u,1,k}}^{r} & dt_{P_{u,2,k}}^{r} \end{array} & \begin{array}{cc} dt_{L_{u,1,k}}^{r} & dt_{L_{u,2,k}}^{r} \end{array} \end{array}\right]}^{T},\;A_{k}$ is the corresponding design matrix taken from Equation (21). $\;\varepsilon_{k}\;$ is observation noise vector and its covariance matrix is $Q_{y_{k}}$. $\Phi_{k,k-1}$ is the state transition matrix. $w_{k}$ is the state translation noise vector and $Q_{w_{k}}$ is its covariance matrix.

The Kalman filter procedure to this equation system can be divided into two parts. One is a standard Kalman filter procedure. The other, as an additional step, is to update the solution from the standard Kalman filter by applying the absolute constraints. The sequential solutions start with the standard Kalman filter with the following equations [37]:

(24a) $${\tilde{x}}_{k}=\Phi_{k,k-1}{\hat{x}}_{k-1}$$

(24b) $$Q_{{\tilde{x}}_{k}}=\Phi_{k,k-1}Q_{{\hat{x}}_{k}}\Phi_{k,k-1}^{T}+Q_{w_{k}}$$

(24c) $$J_{k}=Q_{{\tilde{x}}_{k}}A_{k}^{T}{\left(A_{k}Q_{{\tilde{x}}_{k}}A_{k}^{T}+Q_{y_{k}}\right)}^{-1}$$

(24d) $${\hat{x}}_{k}={\tilde{x}}_{k}+J_{k}\left(y_{k}-A_{k}{\tilde{x}}_{k}\right)$$

$$Q_{{\hat{x}}_{k}}=\left(I-J_{k}A_{k}\right)Q_{{\tilde{x}}_{k}}$$

where $\;{\tilde{x}}_{k}$ denotes the predicted values of the unkowns and $Q_{{\tilde{x}}_{k}}\;$ is its respective covariance matrix. If the tropospheric and ionospheric constraints are available, the additional step is carried out to update the filter solution from the standard Kalman filter [23]:

(25a) $${\hat{x}}_{k}：={\hat{x}}_{k}+Q_{{\hat{x}}_{k}{\hat{\varsigma }}_{k}}{\left(Q_{{\hat{\varsigma }}_{k}}+Q_{\varsigma_{k}^{0}}\right)}^{-1}\left(\varsigma_{k}^{0}-{\hat{\varsigma }}_{k}\right)$$

(25b) $$Q_{{\hat{x}}_{k}}：=Q_{{\hat{x}}_{k}}-Q_{{\hat{x}}_{k}{\hat{\varsigma }}_{k}}{\left(Q_{{\hat{\varsigma }}_{k}}+Q_{\varsigma_{k}^{0}}\right)}^{\text{-}1}Q_{{\hat{\varsigma }}_{k}{\hat{x}}_{k}}$$

We denote the updated solutions using the same symbols as for standard Kalman filter solutions. $Q_{{\hat{x}}_{k}{\hat{\varsigma }}_{k}}\;$ and $Q_{{\hat{\varsigma }}_{k}}$ can be obtained from $Q_{{\hat{x}}_{k}}$.

## 6. Experiments and Results

**Figure 2:**
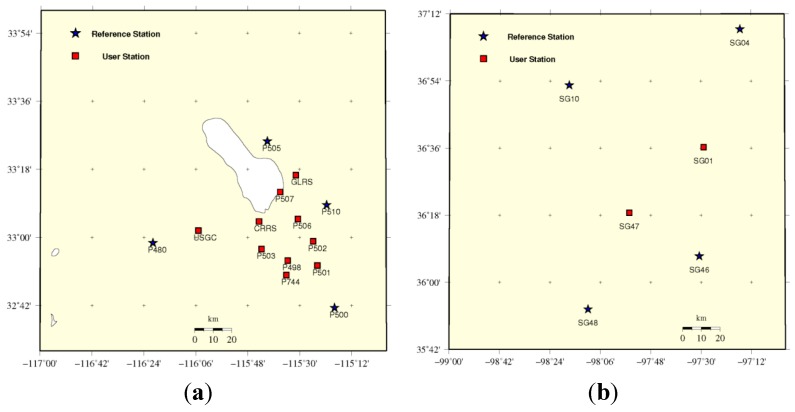
Medium (**a**) and long (**b**) inter-station distance reference network, the red square-user station, blue star-reference station.

Two test networks of different spatial extent are analyzed, one with medium inter-station distances of 41.4–65.3 km and the other with longer inter-station distances of 65.2–114.7 km. The test networks are constructed by using several GPS stations of the USA Continuously Operating Reference Stations (CORS, Figure 2). Ten stations located inside the network with medium inter-station distances were chosen as user receivers. Two stations inside the network with long inter-station distances are selected as user receivers. The observation interval is also 15 s. The elevation cut-off angle is set to 10°. The satellites FCBs are computed using a regional network with 28 stations in North America on Day Of Year (DOY) 3, 2013 in order to have a better fit to the region [22]. The IGS final orbit and clocks are used in the experiment.

Four schemes for determining the variance of ionospheric delays are used for the purpose of comparison. Scheme 1 treats atmospheric correction as deterministic quantities. Scheme 2 uses the accuracy of ZTD $\sigma_{\tau_{k}\;}$ = 1.5 cm recommended by [34] and the accuracy of all the slant ionospheric corrections computed by using the linear function with β = 0.57 mm/km [35]. Scheme 3 uses the accuracy of ZTD $\sigma_{\tau_{k}\;}$ = 1.5 cm and the accuracy of all the slant ionospheric corrections computed by using the linear function with β = 0.74 mm/km [36]. Scheme 4 uses the estimated ZTD and ionospheric accuracies that adaptively varies with the network.

The true ionospheric delays and ZTD at a user station, which is used for assessing the interpolated corrections, can be computed by processing user station as a reference station. The real accuracies of these atmospheric corrections are represented as the difference between the true and interpolated atmospheric corrections. The estimated accuracies of atmospheric corrections can be derived from the covariance matrix determined with Equations (12)–(20).

The new version of the LAMBDA software is applied to conduct partial ambiguity resolution. Both ratio test and success rate are applied to validate the ambiguities. The ratio threshold value is taken w.r.t different ambiguity dimensions as 2 for 2-dimension, 1.5 for 3/4-dimension, 1.3 for 5 to 7-dimension and 1.2 for higher than 7-dimension [23]. The success probability applied is 99.99%. We reinitialize the processing every 5 min for medium network and 10 min for larger network. The ambiguities are fixed only when the thresholds of ratio and success probability are both reached. The fix rate and success rate are defined as:

(26) $$P_{f}=\frac{\#\text{ fixed ambiguities}}{\#\text{ total ambiguities}}\text{ and }P_{s}=\frac{\#\text{ correctly fixed ambiguities}}{\#\text{ total ambiguities}}$$

As claimed in Section 4, the correlations between interpolated ZTDs and ionospheric errors are ignored when we use them at the user stations. To verify that the correlations between interpolated ZTDs and ionospheric errors are small enough to be ignored, we numerically computed the correlation coefficients and tested the hypothesis of no correlation. 

**Figure 3:**
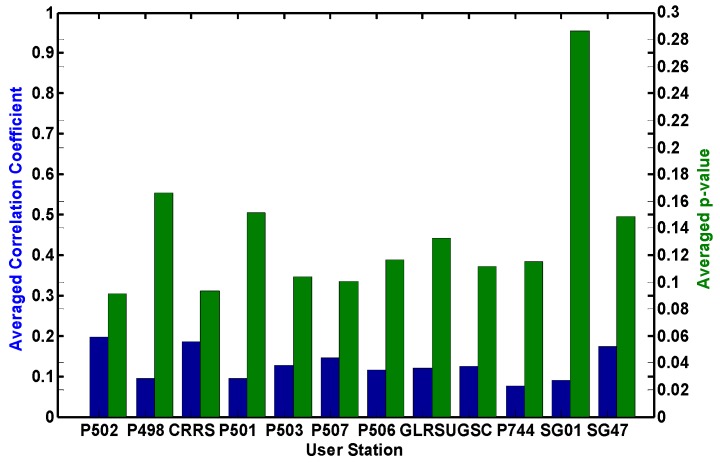
Average correlation coefficient between ZTD and slant ionospheric delays at user stations and p-vales for testing the hypothesis of no correlation.

As shown in Figure 3, the actual correlation between the interpolated ZTD and ionospheric errors is very weak with a mean correlation coefficient of about 0.12 at 12 user stations. Meanwhile, all p-values computed for testing the hypothesis of no correlation are larger than 0.05. Therefore, the correlation is statistically insignificant.

### 6.1. Medium Inter-Station Distance Network

Taking the undifferenced ionospheric and tropospheric delays retrieved at the four reference stations, the accuracies of atmospheric correction determined by different schemes are presented in Table 1. The real accuracies of ZTDs for user stations are from 0.2 cm to 1.4 cm with a mean of 0.8 cm, which essentially reflect the average spatial extent between user station and reference stations. The accuracies of ZTDs using Scheme 4 are from 0.8 cm to 1.0 cm with s mean of 0.9 cm. The results confirm the accuracy of ZTDs using Scheme 4 are consistent with the real accuracies overall except stations P504, GLRS and URGC. The inconsistencies may be caused by the longer distances to reference stations or the bad distribution of the user station.

**Table 1:** The average accuracies of the interpolated atmospheric correction with medium inter-station distance network.

User Station	Ave Dist (km)	$\sigma_{\tau }$ (cm) Real/Scheme 4	$\sigma_{\iota }$ (cm) Real/Scheme 2/Scheme 3/Scheme 4
P502	42.7	1.2/1.0	2.2/2.4/3.2/2.3
P498	46.5	0.9/1.0	2.5/2.7/3.4/2.3
CRRS	42.3	1.3/1.0	1.8/ 2.4/3.1/2.0
P501	47.6	1.0/0.9	3.3/2.7 /3.5/3.4
P503	58.6	0.3/0.9	2.3/3.3/4.3/2.8
P507	37.7	1.4/0.9	1.8/2.1 2.8/1.9
P506	38.9	1.2/0.9	2.2/ 2.2/2.9/2.7
GLRS	38.8	0.2/0.8	3.5/2.2 /2.9/3.6
UGSC	49.5	0.2/0.8	3.5/2.8/3.7/3.4
P744	49.6	0.5/0.8	3.2/2.8 /3.7/3.4

The real accuracies of interpolated ionospheric errors reach 1.8–3.5 cm with mean of 2.8 cm. The accuracies of ionospheric errors determined using the Schemes 2–4 are from 2.1 to 2.8 cm with the mean of 2.6 cm, 2.8–3.7 cm with the mean of 3.4 cm and 1.9–3.6 cm with mean of 2.6 cm. It can be seen that both Schemes 2 and 4 better represent the real ionospheric errors over all.

**Figure 4:**
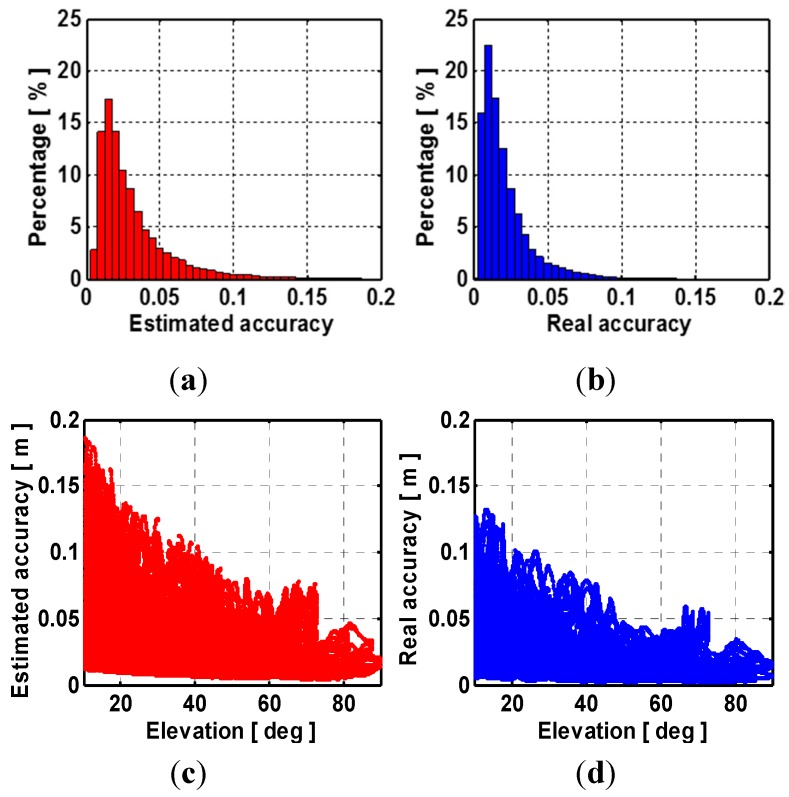
(**a**) distribution of peak-to-peak amplitudes of estimated accuracy of ionospheric correction; (**b**) distribution of peak-to-peak amplitudes of real accuracy of ionospheric correction; (**c**) peak-to-peak amplitudes of estimated accuracy of interpolated ionospheric corrections against elevation function; (**d**) peak-to-peak amplitudes of real accuracy of interpolated ionospheric corrections against elevation function.

**Figure 5:**
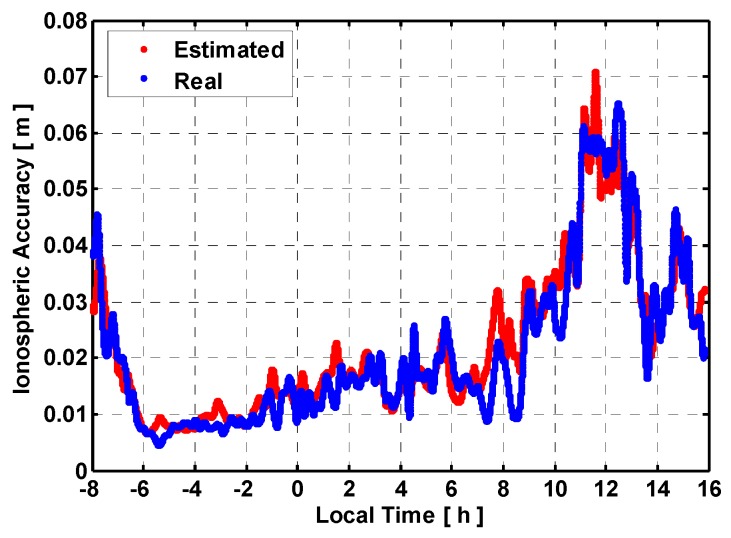
Interpolated slant ionospheric accuracy at user station P502.

For a user station with an average distance of 45 km to the reference stations, the empirical accuracies of interpolated ionospheric corrections determined by Scheme 2 and Scheme 3 are 2.6 cm and 3.3 cm during one day. However, these values are not realistic for all real situation. Figure 4 shows the peak-to-peak amplitudes of both estimated and real accuracies of ionospheric corrections over 24 h against the satellite elevation for all user stations. The estimated and real accuracies of ionospheric correction are shown as red and blue. It can be seen the real accuracy of interpolated ionospheric correction degrades as satellite elevation deceases. Besides, significant ionospheric variation from time to time during one day can be seen from Figure 5. Although empirical accuracies basically reflect the overall size of the ionospheric corrections, it fails to capture these complicated ionospheric variability which differs from time to time and elevation during one day. The estimated accuracy of ionospheric correction using Scheme 4 reflects the overall size of the ionospheric corrections but also capture the ionospheric temporal variability as shown in Figure 5.

**Figure 6:**
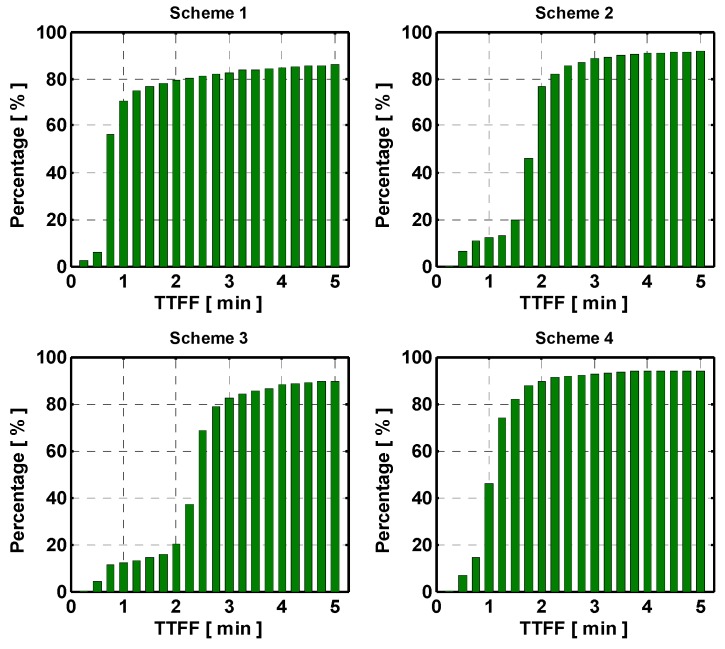
Cumulative distribution of TTFF of the network with medium inter-station distance. The panels from top-left to bottom-right indicate the results with respect to Schemes 1 to 4.

For the medium inter-station distance network, the cumulative distributions of time-to-first-fixed (TTFF), defined as the time needed for successful AR are shown in Figure 6.

The corresponding empirical fix rate and success rate with different schemes are shown in Table 2. 

**Table 2:** 5-min fix rate (*P_f_* ) and success rate (*P_s_*) with different schemes.

User Station	Scheme 1	Scheme 2	Scheme 3	Scheme 4
	*P_f_* *Ps*	*P_f_* *Ps*	*P_f_* *Ps*	*P_f_* *Ps*
P502	98.23 90.71	97.11 93.24	94.28 89.64	98.47 95.08
P498	94.87 87.60	97.26 93.38	97.26 92.47	98.87 95.46
CRRS	95.54 88.22	94.01 90.27	93.90 89.27	95.19 91.91
P501	96.79 89.37	94.78 91.00	95.81 91.09	98.51 95.12
P503	92.22 85.15	96.05 92.22	93.79 89.17	97.97 94.60
P507	95.46 88.15	99.53 95.57	96.68 91.92	98.37 94.98
P506	97.90 90.40	96.67 92.82	96.15 91.41	98.54 95.15
GLRS	88.33 81.56	94.58 90.82	90.25 85.81	93.80 90.57
UGSC	95.65 82.39	94.85 91.07	92.51 87.96	93.95 90.72
P744	92.88 85.77	90.08 86.49	89.96 85.53	92.77 89.57
Average	94.14 86.93	95.49 91.69	94.06 89.43	96.64 93.32

The results reveal that Scheme 4 obtains the highest success rate of 93.3% with 5-min observation accumulation. Scheme 1 fixes most of ambiguities faster compared to the other schemes, as 77.2% of the ambiguities can be fixed within 1 min. After 5 min, the fix rate reaches 94.1%. However, the success fix rate is only 86.9%, which indicates unreliable ambiguity fixing. As to Schemes 2 and 3, the success rates of 5-min solution are improved to 91.7% and 89.3% by empirically considering the stochastic model of atmospheric correction. With the estimated stochastic model of atmospheric corrections, the success rate can be further improved to 93.3% with Scheme 4. Most importantly, Scheme 4 achieves the lowest incorrect fix rate with 3.3%.

### 6.2. Long Inter-Station Distance Network

Table 3 shows the average accuracies of interpolated ionospheric and ZTD corrections for the network with average distance of more than 70 km. 

**Table 3:** Interpolated atmospheric correction accuracy with long inter-station distance network.

User Station	Ave. Dist. (km)	$\sigma_{\tau }$ (cm) Real/Scheme 4	$\sigma_{\iota }$ (cm) Real/Scheme 2/Scheme 3/Scheme 4
SG01	73.7	0.4/1.0	3.1/4.2/5.4/3.2
SG47	73.5	1.1/1.1	4.1/4.2/5.4/5.0

**Figure 7:**
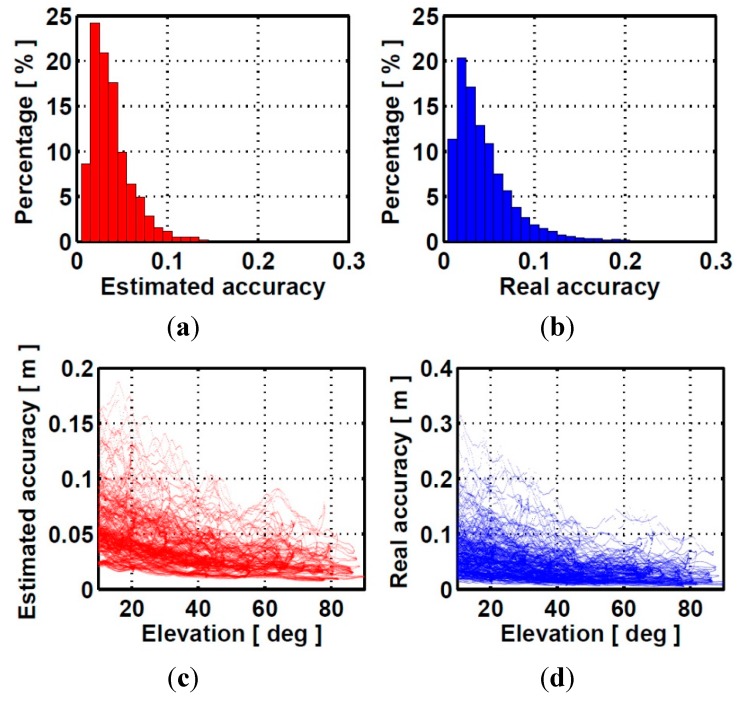
(**a**) distribution of peak-to-peak amplitudes of estimated accuracy of ionospheric correction; (**b**) distribution of peak-to-peak amplitudes of real accuracy of ionospheric correction; (**c**) peak-to-peak amplitudes of estimated accuracy of interpolated ionospheric corrections against elevation function; (**d**) peak-to-peak amplitudes of real accuracy of interpolated ionospheric corrections against elevation function.

**Figure 8:**
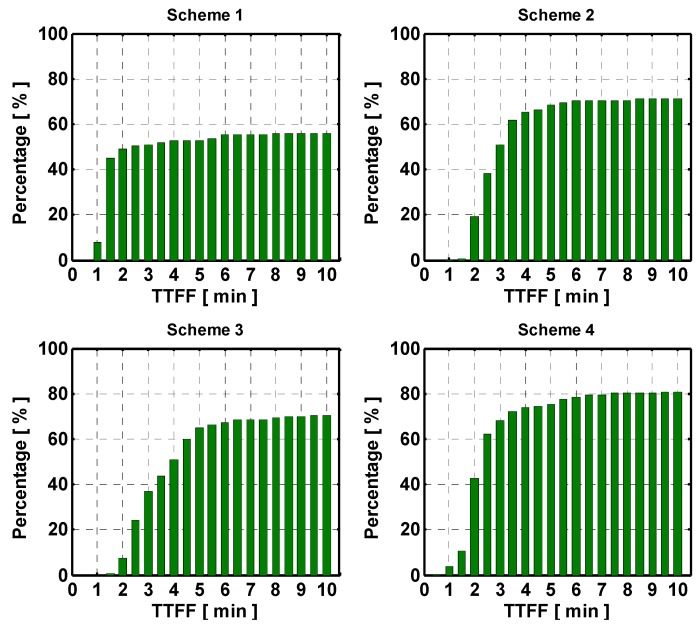
Cumulative distribution of TTFF of the network with long inter-station distance. The panels from top-left to bottom-right indicate the results with respect to Schemes 1 to 4.

The interpolated ZTD errors still remain smaller than 1.5 cm. The interpolated ionospheric correction error become larger compared to those using a medium inter-station distance network. The accuracy of the ionospheric corrections by using Schemes 4 and 3 is the most close to the actual one for stations SG01 and SG47. Figure 7 shows the true and estimated accuracies of the interpolated ionospheric delay with satellite elevation. Similar accuracy degradation with elevation as shown in Figure 4 can be observed. For the long inter-station distance network, the cumulative distributions of TTFFs with different schemes are shown in Figure 8. 

It can be seen that lower success rates are obtained compared to the medium inter-station distance network. This is because larger variances of atmospheric corrections have been assigned to reduce the model strength and subsequently to avoid potential biases in the float solution. As a result, Scheme 1 can successfully fix 49.0% of ambiguities with 2-min observation time. 19.4%, 9.7% and 42.8% of ambiguities can be fixed using Schemes 2–4 with the same observation period. However, the success rate with scheme 1 does not significantly increase with increase of the observation time as those with other schemes. This is because that Scheme 1 treats ionospheric correction as deterministic. Scheme 1 can obtain a fixed solution successfully only if the ionospheric corrections are adequately small, but it fails to fix most ambiguities. For the other schemes, the average success rate is the highest for Scheme 4, which is 80.6% with 10 min observation time. The success fix rate, and fix rate with different schemes are shown in Table 4. The results reveal that the highest fix and success fix rates are obtained with the Scheme 4.

**Table 4:** 10-min fix rate (*P_f_* ) and successful rate (*P_s_*) with different schemes.

User Station	Scheme 1	Scheme 2	Scheme3	Scheme 4
	*P_f_* *Ps*	*P_f_* *Ps*	*P_f_* *Ps*	*P_f_* *Ps*
SG01	86.32 66.38	86.93 72.67	86.32 73.37	97.87 86.51
SG47	65.01 45.16	85.15 69.82	80.01 67.21	86.74 74.60
Average	75.66 55.77	86.04 71.25	83.16 70.30	92.30 80.55

## 7. Concluding Remarks and Future Work

We have developed a method for regional augmented PPP by using atmospheric corrections and their stochastic model derived from regional reference networks. The experimental results reveal that the proposed method can improve the float solutions so that ambiguities can be fixed quickly and reliably by users within the network coverage. For a medium inter-station distance network, the ZTD correction errors are less than 1 cm on average, while the ionospheric correction errors are less than 3 cm. The performance of the proposed regional augmented PPP can be comparable to NRTK since the ambiguities can be fixed with a success fix rate of 93.3% within 5 min. For the long inter-station distance network, both interpolated ZTD and ionospheric correction errors will increase to 2 cm and 5 cm, respectively. The ambiguity success fix rate with the proposed method suffers a degradation compared to the other schemes, but it still achieves a 80.6% rate. This study has only discussed the stochastic model based on a distance-based linear interpolation model. The development of stochastic models for atmospheric corrections based on the other functional models will be further investigated in future work.
